# Acknowledgment to Reviewers of *Pharmacy* in 2020

**DOI:** 10.3390/pharmacy9010029

**Published:** 2021-01-27

**Authors:** Pharmacy Editorial Office

**Affiliations:** MDPI AG, St. Alban-Anlage 66, 4052 Basel, Switzerland

Peer review is the driving force of journal development, and reviewers are gatekeepers who ensure that *Pharmacy* maintains its standards for the high quality of its published papers. Thanks to the cooperation of our reviewers, in 2020, the median time to first decision was 17 days and the median time to publication was 39 days. The editors would like to express their sincere gratitude to the following reviewers for their precious time and dedication, regardless of whether the papers were finally published:
Abrahamsen, BjarkeBecker, JulieAbrons, Jeanine PBedouch, PierrickAdams, SolomonBehzadi, PayamAdil, MohdBekker, CharlotteAl Rihani, SweilemBeloukas, ApostolosAlderman, ChrisBelousova, VictoriaAllegaert, KarelBenavides, SandraAMIRAL, JeanBenzeroual, Kenza E.Anakin, MeganBerger, JérômeAnderson, ClaireBergmo, Trine StrandAnderson, LorindaBerry, TriciaAndreski, MichaelBetancourt, JoseArakawa, NaokoBindoff, IvanArnet, IsabelleBland, ChristopherAronson, BenBlau, JonathanAshiru-Oredope, DianeBleske, BarryAslanidis, TheodorosBollig, GeorgAtkinson, JeffreyBördlein, ChristophAustin, ZubinBorrelli, Eric PAxon, David RhysBorys, KrzysztofAyers, PhilBosnic-Anticevich, SinthiaAzzopardi, LilianBoussios, StergiosBagiu, Iulia-CristinaBožič, BorutBain, AmieBragazzi, Nicola LuigiBaker, DanialBright, DavidBall, Patrick A.Brock, TinaBarnard, MarieBrown, Dana A.Barnett, NinaBrozović, DaniloBattaglia, YuriBrunetti, LuigiBattistella, MarisaBuda, Valentina OanaBaumgartner, Jennifer L.Bungau, SimonaBeck, Tyler C.Bungau, Simona GabrielaBunnell, KristenCorrea, MaríaBurke, RyanCortese, MirkoBustuchina Vlaicu, MihaelaCortez, ValerieCaballero, PabloCorti, ManuelaCaduff-Janosa, PiaCortjens, BartCain, JeffCory, Theodore J.Capasso, RaffaeleCosby, LouiseCappuccilli, MariaCosseddu, Gian MarioCarrouel, FlorenceCosset, François-LoïcCarvajal, Manuel J.Costa, Ana RitaCasanova, Alfredo GinésCostafreda Salvany, Maria IsabelCasey, TakCostan, Victor-VladCastelino, RonaldCostantini, Veronica P.Caturano, AlfredoCostanzo, MicheleCavé, ChristianCôté, MarcelineCeolotto, GiulioCotelle, PhilippeCeulemans, MichaelCotten, MatthewChaar, BettyCouderc, ThérèseChalmers, LeanneCoulibaly, FasseliChan, VincentCox, Andrea L.Chaudhari, SmrutiCox, RobertChaudhri, VirendraCraig, MorganChauhan, NeerajCrawford, LindseyChen, AledaCreager, HannahChen, TingtingCrepin, ThibautChilds-Kean, LindseyCrisci, ElisaChin, JenniferCrocchiolo, RobertoChurchwell, MariannCroft, LarryCieri-Hutcherson, NicoleCrook, BrianClark, CollinCropp, CherylClemmons, AmberCrown, NatalieCohen, Harold C.Csorba, TiborCole, JessicaCuesta, AlbertoCole, JustinCui, HongyuCompton, AlexCulhane, JamesConaldi, Pier GiulioCullen, BryanConfer, Anthony W.Cun, WeiCong, YingyingCusi, Maria GraziaCongBao, KangCynis, HolgerConnerton, IanCzosnek, HenrykConnolly, MichaelD’Angelo, AlbertoCoombs, KevinDa Costa, Rui M. GilCooper, CallumDa Fonseca, Flavio GuimaraesCooper, RichardDabrowska, KrystynaCorcoran, JenniferDacheux, LaurentCorcoran, Jennifer ADahle, Maria KCork, SusanDalal, VijitDalgaard, Tina SørensenDebadyuti, GhoshDalianis, TinaDeboutte, WardDalmon, AnneDebyser, ZegerDalton, KieranDeCaprio, JamesDaly, JanetDecaro, NicolaDamtie, DebasuDecker, AldoD’Andrea, Marco MariaDeFilippis, Victor R.Darpel, KarinDeim, ZoltánDas, KalyanDel Fresno Sánchez, CarlosDastjerdi, AkbarDel Real, GustavoDatta, SiddharthaDel Valle, LuisDaugrois, Jean-HeinrichDelhon, GustavoDavido, DavidDelhon, Gustavo A.Davidson, AndrewDella Bartola, MicheleDavidson, IritDelputte, PeterDavies, Bryan WilliamDelwart, EricDavis, April D.Demangeat, GérardDavis, David A.Dembowski, JillDavydova, JuliaDeng, XufangDawson, Erica D.Deniziak, StanisławDe Alwis, Ruklanthi (Rukie)Denzin, NicolaiDe Benedictis, PaolaDepaquit, JeromeDe Bernardi Schneider, AdrianoDeRemer, David L.De Breyne, SylvainDering Anderson, AllyDe Chiara, GiovannaDesbiez, CécileDe Clercq, ErikDeshpande, MaithiliDe Francesco, RaffaeleDesprès, PhilippeDe Gascun, Cillian F.Desselberger, UlrichDe Graaf, MirandaDesselle, ShaneDe Groof, AdDestainville, NicolasDe La Pena, MarcosDeStefano, Jefferyde la Torre, Juan C.Deutskens, FabianDe Loof, HansDevaux, PatriciaDe Marco, Maria AlessandraDeviatkin, Andrei A.De Miranda, Joachim R.Dhakal, SantoshDe Noronha, CarlosDhungel, PragyeshDe Oliveira, Paulo RenatoDi Bella, SantinaDe Paola, DomenicoDi Gennaro, FrancescoDe Regge, NickDi Giallonardo, FrancescaDe Rosis, SabinaDi Martino, BarbaraDe Souza, William MarcielDi Micco, SimoneDe Swart, RikDi Nunzio, FrancescaDe Toni, LucaDíaz Mochón, Juan JoséDe Voest, MargaretDiaz, ArturoDe Vos, DanielDiaz-Griffero, FelipeDe Vries, Rory D.Diaz-San Segundo, FaynaDeback, ClaireDick, RobertDiederich, SandraDrebot, MikeDieterle, María EugeniaDrigo, MicheleDietrich, EricDrillien, RobertDietrich, IsabelleDrolet, BarbaraDietrich, UrsulaDrozd, MariolaDietzgen, RalfDrozd, RadoslawDijkman, RonaldDruce, JulianDikeakos, JimmyDrucker, MartinDilles, TinneDrummer, HeidiDimienescu, Oana GabrielaDu, JuanDimitrov, KirilDu, KeDimitrov, Kiril M.Dubé, MathieuDineley, KellyDubovi, EdwardDing, Shou-WeiDucatez, MarietteDiskin, RonDucatez, Mariette F.Divakaruni, SrinivasDuchêne, SebastiánDobbler, GerhardDudley, Jaquelin P.Dodds, W. JeanDudman, Susanne G.Dokland, TerjeDugdale, BenjaminDolei, AntoninaDuke, ElizabethDomanovic, DragoslavDunne, MatthewDomańska-Blicharz, KatarzynaDunowska, MagdalenaDombrovsky, AvivDurand, Guillaume A.Domenech, AnaDürrwald, RalfDomier, LeslieDutartre, HélèneDomingo, CristinaDutch, RebeccaDomingo, MarianoDvorak, CherylDomingo-Calap, PilarDyall, JulieDonadu, MatthewDyall-Smith, MikeDonaire, LiviaDykeman, EricDonald, Claire L.Dziewit, LukaszDonato, CelesteE. Krause, JaneDong, ShengzhangEastwell, Kenneth C.Donyai, ParastouEbert, GregDoore, Sarah M.Ebrahimi, DiakoDopazo, Carlos P.Echevarría, Juan E.Dorotkiewicz-Jach, AgataEcke, ThorstenD’orso, IvanEckels, Kenneth H.Dosoky, NouraEden, John-SebastianDoszpoly, AndorEdenborough, KathrynDoty, JeffreyEdgar, JamesDou, XiaoguangEdlefsen, Paul T.Doublet, VincentEgberink, HermanDouglas, Mark W.Eggers, Christian HDowd, Kimberly A.Eggink, DirkDraime, Juanita A.Egyed, LászlóDražić, MajaEhlers, BernhardEhmann, RosinaEyre, NicholasEhret, Megan J.Faaberg, KayEhrhardt, AnjaFabeni, LaviniaEhrlich, MarceloFablet, ChristelleEichwald, CatherineFaccini, SilviaEid, DeebFadare, Olajide O.El Hadi, HamzaFaita, FrancescoElbadry, Maha AFalendysz, Elisabeth A.Eldar, AviFalkenberg, ShollieElena, CriscuoloFalkenberg, Shollie M.El-Gazzar, Mohamed M.Fan, WenchunElghobashi-Meinhardt, NadiaFan, Yi-ChinEl-Hage, NaziraFarenc, CarineElliman, JenniferFarkas, AttilaElliott, Rohan A.Farkas, SilvieEl-Mayet, FouadFast, MarkElofsson, MikaelFaulk, Christopher D.Elsayed, Ahmed MostafaFaulkner, Geoffrey JohnElshabrawy, Hatem A.Fauver, JosephElshesheny, RabehFavia, GuidoEmam, MehdiFavier, Anne-LaureEmerson, JoannaFehr, AnthonyEndo, HisashiFeiss, Michael G.Engeland, Christine E.Feliziani, FrancescoEngland, MarionFeng, CongEnjuanes, LuisFeng, NingguoEnosi Tuipulotu, DanielFera, DanielaErgunay, KorayFerdinandy, BenceEriko, OhsakiFereres, AlbertoErmonval, MyriamFerguson, BrianErtl, ReinhardFernández, LucíaEschbaumer, MichaelFernandez, MelissaEscribano-Romero, EstelaFernández-Alarcón, ClaudiaEscriu, FernandoFerrara, FrancescaEscudero-Abarca, BlancaFerraz Gonçalves, José AntónioEscudero-Pérez, BeatrizFerreira, FernandoEskelin, KatriFerrer, IsidroEssani, KarimFerrero, DiegoEtebari, KayvanFerrés, MarcelaEtienne, LucieFerriol, InmaculadaEttayebi, KhalilFerron, FrançoisEvani, Shankar JaikishanFerry, TristanEvans, Derek P.Feschotte, CédricEverett, Helen E.Fibriansah, GunturEvermann, JimFichet-Calvet, ElisabethEwans, MatthewFiessler, CorneliaEwing, AdamFiguerola, JordiFilipič, BratkoFrancois, CatherineFinetti-Sialer, Mariella M.François, SarahFinlaison, Deborah S.Frangeul, LionelFiorentini, SimonaFrank, LuizaFischer, CarloFrant, MaciejFischer, MelinaFranzoni, GiuliaFischer, NicoleFraune, SebastianFitzpatrick, BeverlyFreiberg, Alexander N.Fiume, GiuseppeFreimanis, GrahamFlannery, JohnFrentiu, FrancescaFlehmig, BertramFrese, MichaelFleming, DamariusFreuling, ConradFlenniken, MichelleFrias Casas, MarioFlenniken, Michelle L.Froissart, RémyFleury, HervéFrolova, ElenaFlood, MichelleFros, JelkeFlores-Guerrero, Jose L.Frossard, Jean-PierreFocosi, DanieleFrunzke, JuliaFolegatti, PedroFrutos, RogerFoley, Brian T.Fu, Tong-MingFonseca, Gregory J.Fuchs, MarcFonseca, KevinFuji, KevinFontana, JuanFuji, Shin-IchiFontanay, StéphaneFujikura, DaisukeFonzo, MarcoFujita, RyosukeFoppa, Ivo M.Fujiwara, KeiFord II, James H.Fukuhara, TakasukeFord, KathrynFukushi, HidetoForni, DiegoFukuta, ShiroForrest, CraigFukuzawa, Noriho H.Forrester, NaomiFuller, Frederick J.Forsman, MatsFuller, JoanneForstova, JitkaFuller, TrevonFortuna, ClaudiaFurlini, GiulianoForward, SonjaFuruse, YukiForzan, MarioFuruya, TetsuyaFossé, PhilippeFuruya, YoichiFossen, TorgilsFusco, DahleneFoster, JannGabriel, EmmanuelFoster, Toshana LauriaGaffuri, AlessandraFox, JulieGaglia, Marta MariaFoxi, CiprianoGagne, RoderickFrada, MiguelGai, ZhiboFralick, Joe A.Gaitonde, VishwanathFrança, Monique S.Gajda, AnnaFrancis, Ashwanth ChristopherGajdács, MárióFranco, DavidGalabov, AngelGałas, AleksanderGerlich, WolframGale, PatrickGershon, MichaelGallagher, PaulGestal, MonicaGallagher, ThomasGhosh, SouvikGallardo, RodrigoGhosh, SumitGallego, GisselleGiadinis, Nektarios D.Gambardella, ClaudioGiammanco, Giovanni MGambarian, AlexandraGiangaspero, MassimoGambino, GiorgioGiannecchini, SimoneGammon, DonGibuła-Tarłowska, EwaGanaie, SafderGifford, AlisonGandre, CoralieGifford, RobertGanges, LlilianneGil, Jose FernandoGanji, RakeshGill, JasonGann, Eric R.Gimenez-Lirola, Luis G.Ganor, YonatanGinet, NicolasGanser-Pornillos, BarbieGiovanardi, DavideGanser-Pornillos, Barbie K.Gish, RobertGao, YongGiuffrida, PaoloGarbuglia, Anna RosaGladue, DouglasGarcía, PedroGlasa, MiroslavGarcia-Dorival, IsabelGlenn, WendyGarcia-Estañ, Luis PerezGlover, AnthonyGarcía-Murria, María J.Glowacz, AdamGarcia-Ruiz, HernanGoff, DebraGardner, Matthew J.Goffinet, ChristineGarfield, SaraGoh, Gerard Kian-MengGarfinkel, DavidGojani, Ardian B.Garigliany, Mutien-MarieGoldbourt, AmirGarofalo, MariangelaGoldman, JoanneGarten, WolfgangGoldstein, RichardGascuel, OlivierGoldstein, RonaldGastaminza, PabloGolec, PiotrGattinger, PiaGolender, NataliaGaudin, YvesGolender, NatalyaGaudreault, Natasha NGoletti, DeliaGauger, Phillip C.Golubchik, TanyaGavurová, BeátaGolubovskaya, VitaGehrke, LeeGómez Castilla, JordiGeisler, ChristophGomez Monterrey, IsabelGeldenhuys, MarikeGomez-Lucia, EsperanzaGelissen, IngridGong, BinGendrot, MathieuGong, PengGeneroso, Jaqueline Da SilvaGonzales, Jose LGeng, TingtingGonzalez, GabrielGerber, Priscilla F.Gonzalez, Jean-PaulGerhauser, IngoGonzalez, John-PaulGonzalez-Alvarez, MartaGustin, KurtGonzález-Bulnes, AntonioGutiérrez-López, RafaelGonzalez-Dunia, DanielGuy, BrunoGonzalez-Reiche, Ana SilviaGuy, Paul L.González-Zamar, Mariana-DanielaGuydosh, Nicholas R.Goodier, Martin R.Győző, KajánGoodman, Alan G.Gysi, DeisyGoodman, Cynthia L.Gyula, PéterGoodman, Lauren F.Haan, Peter DeGoodwin, ThomasHaase, Astrid D.Gossner, Céline Marie EliseHabili, NuredinGouandjika-Vasilache, IonelaHadidid, AhmedGozalbo Rovira, Roberto VicenteHadziyannis, EmiliaGralinski, LisaHaese, Nicole N.Grand, RogerHagiwara, KatsuroGrandi, NicoleHahm, BumsukGrant, MichaelHahn, AlexanderGraudins, Linda V.Hai, RongGray, Jeffrey A.Hairgrove, ThomasGreen, KimHajarizadeh, BehzadGreen, ToddHakami, RaminGregorio, JoaoHakata, YoshiyukiGregório, JoãoHaley, Sheila A.Greiner, TimoHall, Roy A.Greive, SandraHallden, GunnelGriffin, HeatherHall-Mendelin, SonjaGriffiths, David J.Hamann, KerstinGrimsley, NigelHamel, RodolpheGrose, JulianneHammarskjöld, Marie-LouiseGrose, Julianne H.Hammond, JohnGrove, JoeHampson, LynneGroves, IanHan, Hui-JuGrubman, MarvinHan, LeiGrünweller, ArnoldHAN, ZheGu, ZhenHang, JunGuardado-Calvo, PabloHankaniemi, Minna M.Gudka, SajniHanks, Ephraim M.Guerra-Assuncao, AfonsoHann, Hie-WonGuerrero-Plata, AntonietaHanna, Lezley-AnneGuerrini, MarcoHans, AymericGuharoy, RoyHansel, KatharinaGuido, PoliHaqshenas, GholamrezaGuillaume, MousseauHaque, MuzammelGuillon, ChristopheHarder, TimmGuo, Ming-LeiHardies, Stephen C.Guo, Zong ShengHarding, RobGupta, KapilHarmon, Karen M.Harper, David R.Herker, EvaHarrach, BalázsHernáez, BrunoHarrich, DavidHernández, CarmenHarris, AudrayHernandez, JesusHarris, ReubenHernández, MartaHarrison, RobertHernandez-Vargas, EstebanHarrod, RobertHernandez-Vargas, Esteban A.Hartert, Tina V.Herod, MorganHartshorn, Kevan LHerrera-Carrillo, ElenaHartung, Hans-PeterHersperger, Adam R.Harty, RonaldHertel, RobertHarvey, WilliamHerzog, CatherineHassan, Sharifah SyedHeselpoth, RyanHatano, YuichiroHeslop, IanHatfull, GrahamHess, KarlHattori, ToshioHesson, JennyHause, BenHicks, Julie A.Hawboldt, JohnHijano, Diego R.Hawes, PhilippaHildt, EberhardHay, Iain DHill, DuncanHayasaka, DaisukeHill, FraserHayer, JulietteHillman, LisaHayes, Sidney J.Hillyer, JulianHazan, RonenHilton, Thomas F.He, JieHily, Jean-MichelHe, Mike Z.Hingamp, Pascal M.He, QigaiHinkula, JormaHearing, PatrickHinton, DeborahHébrard, EugénieHipp, KatharinaHedman, KlausHirayama, KazuhiroHefferon, Kathleen L.Hirono, IkuoHefferon, Kathleen LauraHirota, JeremyHegele, Richard GHirsch, Alec J.Heinlein, ManfredHirsch, HansHeitz, ThierryHirsch, IvanHejnar, JiriHirsch, JudithHejnova, LucieHirsch, Silvia AyoraHelbig, KarlaHitch, GeetaHelle, FrancoisHizi, AmnonHenderson, AndrewHo, Chak-SumHenderson, Andrew J.Ho, Eric S.Heng, XiaoHo, Ya-ChiHenke, AndreasHobson-Peters, JodyHenningsen, EmilyHocine, MouniaHenrich, Curtis J.Hodcroft, Emma B.Hepojoki, JussiHodurski, TraceyHerchenröder, OttmarHoeben, RobHoek, Lia Van DerHuang, ZiweiHoenen, ThomasHubai, AndrásHoffmann, MarkusHubal, RobertHöfle, UrsulaHubálovský, ŠtěpánHofstetter, Amelia R.Huber, AndrewHogan, Michael J.Huber, VictorHogue, IanHufbauer, MartinHolbrook, MichaelHufton, Simon E.Holicki, Cora M.Hughes, Holly R.Holland, ChristyHughes, JosephHollingworth, SamanthaHughes, LouiseHollister, Emily B.Hughes, StephenHolmes, Edward C.Hume, AdamHolmes, ErinHummel, MaryHölzer, MartinHunter, TracyHolzhauer, MennoHurley-Kim, KeriHoma, Fred L.Hurst, ChristonHong, XupengHutson, ChristinaHopken, Matthew W.Huynh, ChiHorhat, Florin GeorgeHwang, Eung-SooHorie, MasayukiHynes, AlexanderHorimoto, TaisukeHyodo, KiwamuHorton, NancyHytönen, VesaHorton, Nancy C.Iasevoli, FeliceHorwood, Paul FIboi, Enahoro A.Hosmalin, AnneIbrahim, AhmedHostnik, PeterIbrahim, Maria SalemHou, XiaorongIkebuchi, RyoyoHou, YixuanIkeda, TerumasaHouldcroft, CharlotteIlias, IoannisHoule, SherilynImai, MasakiHouston, FionaImbeault, MichaelHovi, TapaniIncarnato, DannyHraber, PeterIndugu, NagarajuHribar, LawrenceInoue, JunHristov, Peter IvanovIordanskiy, SergeyHsieh, Ming KunIorga, MagdalenaHsieh, Shie-LiangIorio, Ronald M.Hu, JiafenIsabelle, PrêcheurHu, YanmeiIsakova-Sivak, IrinaHuang, ChengIsel, CatherineHuang, ChienjinIseni, FrédéricHuang, Hsiao-LingIshibashi, KazuhiroHuang, Hsing IIshikawa, MasayukiHuang, Jason C.Ishikawa, TomohiroHuang, Yen-MingIshima, RiekoHuang, YongIshizaki, AzumiIskandar, KatiaJayakumar, RebeccaIskra-Caruana, Marie-LineJeffers, MeghanIslam, ArifulJeger, MichaelIto, KimihitoJenckel, MariaIto, NaotoJenkins, FrankIturriza-Gomara, MirenJeonga, Dae GwinIvankovic, TomislavJeske, HolgerIwano, HidetomoJiang, GuochunIwanowicz, Luke RJiang, Qiu-XingIwasaki, MasaharuJiang, ShijinIzsvák, ZsuzsannaJiang, SizunJ. Jackwood, DaralJillella, DineshJääskeläinen, Anne J.Jiménez De Oya, NereidaJackova, AnnaJin, Dong-YanJackson, BrianJin, JingJackson, Kathy M.Jin, TengchuanJackson, Sarah E.Jin, YuefeiJacob, GopasJochmans, DirkJacob, SabrinaJõers, PriitJacobsen, RamuneJohn, Sinu P.Jacobs-Sera, DeborahJohnson, JohnJadhao, Samadhan J.Johnson, NicholasJaguva Vasudevan, Ananda AyyappanJohnson, Reed F.Jaimes, Javier A.Johnston, J. SpencerJakab, FerencJohnston, Randal N.Jakimovski, DejanJończyk-Matysiak, EwaJakobsson, JohanJones, Bryony A.Jalava, KatriJones, ClintonJames, BurkeJones, IanJames, Claire DJones, JeremyJames, DelythJones, MelissaJames, WilliamJordan, BrianJamin, MarcJordan, Jeanne A.Jan, EricJordan, KingJan, Fuh-JyhJordan, Michael RJancovich, JamesJørgensen, Charlotte SværkeJanelle, ValerieJose, JoyceJang, Soo MingJoshi, HirenJangra, RohitJosset, LaurenceJanikowska, GrażynaJulander, Justin G.Janke, KristinJung, AlainJanowski, Andrew B.Jung, Yong SamJansen, C.A. (Christine)Jurak, IgorJansen, StephanieJurvansuu, JaanaJardine, PaulKaae, SusanneJarocki, PiotrKaczmarek, Maria E.Jarosinski, KeithKaczorowski, TadeuszKading, Rebekah C.Kedzierska, KatherineKaelber, JasonKeers, RichardKafri, TalKehn-Hall, KyleneKageyama, TsutomuKeita, AlphaKainulainen, Markus HKelch, BrianKaján, Győző L.Kell, AlisonKajihara, MasahiroKelly, DervlaKalinowski, JörnKelly, FionaKallies, ReneKelly, GabrielleKalra, Raj SinghKemenesi, GáborKälvemark Sporrong, SofiaKennedy, LeanneKamau, EverlynKennedy, Mary-ClaireKanda, TatsuoKennedy, MelissaKanda, TeruKennedy, Richard B.Kandasamy, RamKenney, Joan L.Kandeel, MahmoudKenney, Scott P.Kang, Sang-MooKeramitsoglou, KiriakiKant, SashiKhan, EhsanKantor, AsherKhaperskyy, Denys A.Kaplan, ShaunaKhatri, VishalKapočiūtė-Dzikienė, JurgitaKhayat, RezaKaren, HuhnKheimar, AhmedKarimi, KhalilKhurshid, ZohaibKarlsson, ErikKibenge, FrederickKarniychuk, UladzimirKibler, Karen V.Károly, FátyolKiefer, DorotheeKarsten, Christina BeatriceKiełpińska, JolantaKaruppannan, Anbu KumarKieran, Troy JKashanchi, FatahKierzek, ElzbietaKashiwagi, AkikoKiga, KotaroKathuria, HimanshuKil, Eui-JoonKatneni, UpendraKile, James C.Katoh, HiroshiKilleen, RosemaryKatsoulos, Panagiotis D.Killian, Mary LeaKatz, RichardKilpatrick, MarmKatzenellenbogen, Rachel A.Kim, ChonsaengKaufer, Benedikt B.Kim, Dal YoungKaufmann, AndreasKim, Hye KwonKaul, MarcusKim, In-JeongKautz, Tiffany F.Kim, Jee HyunKawada, Jun-ichiKim, JoomyeongKay, BrianKim, Ki-HongKayali, GhaziKim, MeehyeinKazama, ShinobuKim, MikyeongKe, Po-YuanKim, SeilKearney, Mary F.Kim, Su-miKebodeaux, ClarkKim, SunheeKimpel, JanineKoivisto, JanneKimura, HirokazuKolawole, AbimbolaKincaid, RodneyKolbasov, DenisKinchington, PaulKolesar, Jill M.King, DonaldKolishetti, NageshKing, LindaKomar, NicholasKingsley, DavidKomatsu, KenKipar, AnjaKomorowska, BeataKirchhoff, FrankKondili, LoretaKireev, DmitryKondo, HidekiKirk, LorenKondo, SatoruKirkwood, CarlKönig, AlexanderKirubakaran, PalaniKonjevic, DeanKirui, JamesKoodie, LisaKis, ZoltanKoos, BjörnKiseleva, Irina V.Kordyukova, LarisaKishida, YutakaKormelink, RichardKiso, MakiKormuth, KarenKitchen, AndrewKoromyslova, AnnaKlapper, PaulKorth, JohannesKlase, Zachary A.Koshiba, TakumiKlein, Terry A.Koshizuka, TetsuoKleinow, TatjanaKosik, IvanKleinpeter, AlexKoskan, AlexisKlenk, Hans-DieterKoslová, AnnaKlichowski, MichalKostaki, EvangeliaKlimkait, ThomasKoster, AndriesKlímová, BlankaKostyushev, DmitryKlonjkowski, BernardKosulin, KarinKlose, ThomasKotani, KazuhikoKlyczek, KarenKotta-Loizou, IolyKnapp, Katherine K.Kouyos, Roger DKnepper, JaniceKovarik, AlesKnepper, ToddKovvasu, Surya PrakasaraoKnight, Richard J.Kozak, ChristineKnowles, GraemeKozieł, EdmundKnowles, Nick J.Koziol-White, Cynthia J.Ko, Eun-juKozlovskaya, LiubovKobayashi, TetsuroKozlowski, LukaszKobie, James J.Krammer, FlorianKobiler, OrenKraska, ThorstenKochneva, GalinaKrejbich-Trotot, PascaleKock, RichardKrenz, BjörnKodidela, SunithaKrishnakumar, DevadasKoehler, TamaraKrishnamurthy, Siddharth R.Koethe, SusanneKroeker, AndreaKohara, MichinoriKrok, DariuszKrol, EwelinaKwon, AmyKroon, LisaKwon, SunohKrug, LaurieKwun, Hyun JinKrug, PeterKyei, George BKruger, DetlevL. Schmaljohn, AlanKrüger, NadineLa Rosa, GiuseppinaKruse, Robert L.Laanto, ElinaKryczyk-Poprawa, AgataLabo, NazzarenaKryger, PerLacey, Charles J. N.Krylov, VictorLagatolla, CristinaKrzystanek, MarekLagaudrière-Gesbert, CécileKrzyzowska, MalgorzataLagunoff, MichaelKubo, YoshinaoLai, Michael M. C.Kubota, ToshioLaksono, BrigittaKucheryavykh, Lilia Y.Lamb, ChristopherKugelman, Jeffrey R.Lamb, LauraKuhar, UrškaLamba, DorianoKula-Pacurar, AnnaLamberton, NathanKulkarni, AmolLaMere, SarahKumar Kundu, JibanLanave, GianvitoKumar, AmitLang, AndrewKumar, AshokLang, FengchaoKumar, AsitLange, UlrikeKumar, BinodLangel, Stephanie NealKumar, MukeshLangsjoen, RoseKumar, PenmetchaLanteri, GiovanniKumar, PraveenLantta, TellaKumar, ShaileshLarussa, TizianaKumari, AshaLattorff, H. Michael G.Kümmerer, Beate M.Lauzi, StefaniaKunec, DusanLaval, KathlynKuniya, ToshikazuLaw, AnandiKuo, Yen-WenLawrence, Charles M.Kurebayashi, YuukiLazar, CatalinKurian, Justin J.Le Goffic, RonanKurt, ToblerLe Mercier, PhilippeKüry, PatrickLe Pendu, JacquesKuryk, LukaszLe Sage, ValerieKusejko, KatharinaLeache, LeireKuss-Duerkop, Sharon K.Leal, Ana MendesKuzmak, JacekLean, Fabian Z. X.Kuzmin, IvanLebrand, AitanaKuzmina, Natalia A.Lednicky, JohnKvale, DagLee, JaehwiKwak, Sang HoLee, Joo-YounKwiatek, OlivierLee, KellyKwilas, StevenLee, Keun HwaLee, Kuo HaoLi, XiangminLee, Min YoungLi, XiaojunLee, Tzong-ShyuanLi, YanliLeeks, AsherLi, ZhuoranLeeuw, Erik DeLiang, ChenLefkowitz, Elliot J.Liang, Po-HuangLegendre, MathieuLiang, YuyingLegler, PatriciaLiao, JiawenLehman, Dara A.Liao, Laura E.Lehman, John M.Liao, Yu-ChiehLeifels, MatsLiberski, Paweł P.Leis, Jonathan P.Lichterfeld, MathiasLeisi, RemoLim, Chun ShenLeitão, AlexandreLim, Siew PhengLeite, Silvana NairLim, Stephanie MLemay, GuyLin, Chao-NanLemmermann, Niels A WLin, Cheng-WenLenz, OndřejLin, TaoLeon-Juarez, MoisesLindberg, A. MichaelLequime, SebastianLindemann, DirkLeroux, CarolineLindenbach, BrettLesbats, PaulLing, ChenLester, Philip JohnLing, HuaLetarov, AndreyLing, JiaxinLetoha, TamásLing, JiqiangLeung, AnthonyLingwood, DanielLeung, Daisy W.Linial, MaxineLeung, Kwong-SakLinial, Maxine L.Leutenegger, Christian M.Lipińska, AndreaLevêque, DominiqueLittlejohn, MargaretLevin, JudithLiu, GuanQunLevrero, MassimoLiu, HaolinLevy, Ronald M.Liu, Hsin-FuLewis, SusanLiu, JiaLewitus, EricLiu, QiangLeymarie, OlivierLiu, Shan-LuLhomme, SébastienLiu, WeiLi, ChunfengLiu, XiaojieLi, DapengLiu, XiaoyingLi, HaiLiu, YueLi, HongminLiu, ZhuomingLi, JinquanLizano, MarcelaLi, JonathanLizarazo, Erley F.Li, LinLlanes, AlejandroLi, Melody HingLlanes, CarlosLi, RenfengLlorente, FranciscoLi, ShitaoLo Giudice, RobertoLo, Shih-YenLycett, SamanthaLo, SzechengLynch, RebeccaLoc-Carrillo, CatherineLynch, SarahLodmell, J. StephenLynch, Stacey ELogan, PatriciaLyons, KayleyLogue, JamesLyu, YuanLohmann, VolkerMa, JiaLojkić, IvanaMa, WenjunLondon, Robert E.Mabashi-Asazuma, HideakiLondono, BerlinMacchi, BeatriceLondrigan, SarahMacdonald, AndrewLong, Chiau MingMacDuff, Donna A.Lopes, Daniel SimõesMacfarlan, ToddLópez, Carolina B.Machado, AlexandreLópez, DanielMachová, KristínaLopez-Ferber, MiguelMaciejewska, BarbaraLópez-Ferber, MiguelMaciver, SutherlandLorenzo, GemaMackler, EmilyLorusso, AlessioMacLure, KatieLos, MarcinMacNeill, AmyLotz, JeffreyMaeda, KazuhikoLouis, Irina St.Maeda, NaoyoshiLovell, ScottMaekawa, ShunLowe, DavidMager, Dixie L.Lozach, Pierre-YvesMaggi, FabrizioLozano, Jose ManuelMaggirwar, SanjayLozano-Durán, RosaMaghsoodi, AmenehLu, TzupinMaginnis, MelissaLucas, CherieMagor, KatharineLucena, MarianaMahar, JackieLucifora, JulieMahony, Timothy JohnLuetsch, KarenMaia, MariaLühken, RenkeMaillard, Pierre VLukacher, AronMaisetta, GiuseppantonioLukashevich, IgorMaixner, MichaelLukhovitskaya, NinaMajerciak, VladimirLulla, ValeriaMajewski, JacekLundgren, MagnusMajkowska-Skrobek, GrażynaLundstrom, KennethMak, Lung-YiLuo, ZhenMakarova, Kira SLupberger, JoachimMakowski, LeeLusardi, KatherineMalagon, FranciscoLüscher, BernhardMaldarelli, FrankLustig, YanivMaldonado, JuanLuthra, PriyaMalekigorji, MaryamLuttermann, ChristineMalhotra, JodieLy, HinhMalone, DanielMamede, JoaoMartinez-Gil, LuisManchia, MirkoMartínez-Martínez, IreneMang, Stefania MirelaMartini, Nataly DominicaMangala Prasad, VidyaMartins, NelsonMangino, GiorgioMarty, GaryManicassamy, BalajiMarutescu, LuminitaMann, ClaireMarutescu, Luminita GabrielaMans, JanetMaruyama, JunkiMantzourani, EfiMarzano, Shin-YiManuel Giorgi, FedericoMas, AntonioManzoor, RashidMasalova, Olga V.Marc, DanielMasataka, TsugeMarcacci, MauriliaMassanella, MartaMarchant, DavidMassimi, AzzurraMarchetti, MoniaMastino, AntonioMarchini, AntonioMasuda, TakaoMarchiò, SerenaMatalka, Khalid Z.Marenzoni, Maria LuisaMatczuk, Anna KarolinaMargina, DenisaMateo, MathieuMargulies, BarryMateu-de Antonio, JavierMarin Lopez, AlejandroMathew, AnujaMariner, JeffreyMathieson, StephanieMaringer, KevinMathieu, CyrilleMarjomaki, VarpuMathijs, ElisabethMark S., JohnsonMatsuo, EikoMarkovich, Michal PerryMatsushita, MichikoMarkowicz-Piasecka, MagdalenaMattapallil, JosephMarkussen, TurhanMatthews, LouiseMarquet, RolandMattingly, AshleeMarriott, AnthonyMattingly, JoeyMarschall, ManfredMattsson, SofiaMarsden, MatthewMatusali, GiuliaMarston, DeniseMauffret, OlivierMarszałek, JarosławMaury, MarcMartella, VitoMavian, CarlaMartikainen, MiikaMay, JaredMartín Castillo, MargaritaMay, MeganMartin, AnnetteMaynard, GreggoryMartin, EileenMazurov, Dmitriy VMartin, JavierMazzei, MaurizioMartin, StephenMbonye, UriMartin, Tammy M.McAuley, JamesMartín-Acebes, Miguel A.McBane, SarahMartinet, Jean-PhilippeMcBride, JohnMartinez, Jose CMcCarrell, JamieMartínez, Miguel A.McCauley, MicahMartínez-García, ManuelMcCormick, CraigMcCulloch, KarenMetifiot, MathieuMcDonnel, Samantha J.Metz, StefanMcFadden, GrantMetz, Stefan W.McFarlane, Donald A.Metzger, MichaelMcGee, Edo-abasi U.Metzner, KarinMcIlroy, DorianMeurens, FrancoisMcInerney, GeraldMeurs, ElianeMcKee, CliftonMeuti, MeganMcKeirnan, KimberlyMeyer, FlorenciaMcknight, AineMeyer, GillesMcLean, GaryMeyer, HermannMcLean, RebeccaMeyer, JustinMcMillan, NigelMeyers, CraigMcVey, ColinMiao, QiuhongMeaney, Calvin J.Michaux, J. R.Medina Piles, VicenteMichon, ThierryMedina, Gisselle N.Miesen, PascalMedina-Kauwe, Lali K.Milani, AdelaideMediouni, SoniaMilani, MarioMee, Edward T.Milavetz, BarryMeertens, LaurentMillard, AndrewMehedi, MasfiqueMillard, Andrew D.Mehta, Sanjay R.Miller, W. AllenMei, Ya-FangMiller-Saunders, KristiMeier-Stephenson, VanessaMillet, Jean K.Melcher, UlrichMinematsu, ToshioMelikian, GregoryMinshew, LanaMeliopoulos, Victoria AMira, José JoaquínMelo, PedroMirabelli, CarmenMelzer, Michael J.Miranda, CarlaMena, IgnacioMiroslaw, SnieturaMencia Caballero, MarioMishra, AwdheshMendoza, EmelissaMiskulin, IvanMenéndez-Arias, LuisMitsuru, OkuwakiMeng, XiangchaoMixon, AmandaMenne, StephanMiyashita, ShuheiMenzo, StefanoMiyazawa, MasaakiMeredith, AshleyMizukami, ShusakuMergia, AyalewMlera, LuwanikaMerino, Jose JoaquinMocarski, EdwardMerkling, Sarah HeleneMochizuki, TomofumiMesci, PinarModahl, CassandraMeshram, ChetanModha, SejalMesko, MajaMoens, Ugo LionelMeškys, RolandasMognetti, BarbaraMesri, EnriqueMohanty, SmitaMestres, ConxitaMöhl, BrittaMohr, Emma L.Mukhopadhyay, TuliMohsen, MonaMulder, LubbertusMok, Chris C.P.Mulkey, SarahMolina-Mula, JesúsMullarkey, CaitlinMolineux, IanMüller, BarbaraMonaghan, ThomasMuller, David AlexanderMonguió-Tortajada, MartaMüller, JanisMonne, IsabellaMuller, MandyMontagutełłi, XavierMuluneh, BenyamMontassier, Helio J.Munk, CarstenMontes, NuriaMuñoz-Barroso, IsabelMontoya, MariaMünstedt, KarstenMoore, GinaMuntean, Alexandru AndreiMoore, Nicholas M.Munusamy, ShankarMoore, PatrickMurakami, HironobuMorabito, Kaitlyn M.Murakami, KeisukeMoraes, TheoMurakami, KosukeMorais, MarcMurakami, ShinMoraru, Liliana CristinaMurakami, YoshikiMordecai, GideonMuramatsu, MasamichiMoreno, MiguelMuraru, Iulia-DianaMorgado, ManuelMurata, TakayukiMorgan, EthanMuriaux, DelphineMorgan, Ethan L.Murillo, RosaMorgan, JillMurota, KatsunoriMori, YasukoMurovska, ModraMorimoto, KinjiroMurovska, Modra F.Morita, EijiMurphy, Alex MMorley, ValerieMurphy, EainMorozov, IgorMurray, Shannon M.Morozova, OlgaMusalgaonkar, SharmishthaMorozova, Vera V.Musk, BillMorris, ColetteMustafa, GhazalaMorris, MhairiMuthu Karuppan, Mohan KumarMorrison, JulietMutoloki, StephenMorrison, Thomas E. “Tem”Muxel, Sandra MarciaMorrissey, HanaMyers, DeanMospan, CortneyMylrea, MartinaMoss, BernardMyung, HeejoonMossel, EricNachbagauer, RaffaelMostafa, AhmedNaesens, LieveMotamed, CyrusNagafuchi, SeihoMousa, Jarrod J.Nagai, MakotoMoutailler, SaraNagasaka, KazunoriMoutelíková, RomanaNaghavi, Mojgan H.Mucker, Eric M.Naguib, MahmoudMühlberger, ElkeNaik, ShruthiNaji, Hassan S.Nishizono, AkiraNakagawa, KojiNisole, SébastienNakagawa, SoNissimov, JozefNakahara, Kenji S.Nistal-Villán, EstanislaoNakahara, TomomiNjeumi, FelixNakakita, Shin-ichiNofemela, Robert S.Nakanishi, AkiraNogales, AitorNakauchi, MinaNogalski, MaciejNakayama, EriNoguchi, YoshihiroNakayashiki, HitoshiNogueira, MauricioNakazawa, YoshinoriNoris, EmanuelaNanbo, AsukaNorman, Wendy V.Narayan, SujitaNovacek, JiriNasar, FarooqNovelli, GiuseppeNash, CarolNovosel, DinkoNaughton, BernardNowotny, NorbertNavarro, BeatrizNuesch, JurgNavas-Martin, SoniaNüesch, JürgNavratil, VincentNugen, SamNawrocki, Steffan T.Nunberg, JackNawrot, RobertNunez, AlejandroNayak, TapanNúñez, José IgnacioNazar, HamdeObreza, AlešNazli, AishaObuobi, Sybil Akua OkyerewaNeerukonda, SabarinathO’Connor, ChristineNejman-Faleńczyk, BożenaOde, HirotakaNekhai, SergeiOdendall, CharlotteNelson, Scott W.Oesterle, PaulNemecek, BrandenOfferdahl, Danielle K.Nemes, KatalinOgasawara, NorikoNerva, LucaOgden, KristenNetherton, Christopher L.Ogembo, JavierNeufeldt, Christopher JohnOgino, TomoakiNeuman, BenjaminOhshima, TakayukiNeumann, DonnaOka, TomoichiroNevola, RiccardoOkabayashi, TamakiNice, TimothyOkamoto, HiroakiNicoletti, LoredanaOkamoto, ShigefumiNiedźwiedzka-Rystwej, PaulinaOkayama, AkihikoNielsen, Tue KjærgaardOkazaki, KatsunoriNiepmann, MichaelOksanen, HannaNiespodziana, KatarzynaOlagoke, OlusolaNigam, DeeptiOlds, Cassandra L.Niikura, MasahiroOlech, MonikaNiquille, AnneOlejniczak, MartaNishigaki, KazuoOlejnik-Schmidt, AgnieszkaNishihara, HidenoriOlety, BalajiOliver, JoAnnePandit, AridamanOlson, AnthonyPanfil, AmandaOlson, KennethPanganiban, AntonitoOng, David S.Y.Panicz, RemigiuszOnomoto, KojiPantůček, RomanOomens, Antonius (Tom)Papin, James F.Opekun, Antone RobertPappu, HanuOrchard, RobertPaquin-Proulx, DominicOrlando, ValentinaParadkar, PrasadOrlova, Elena V.Parajuli, Daya RamOrłowska, AnnaParan, NirOrnelles, DavidParcells, MarkOrrú, ChristinaParikh, MamtaOrtega, Joseph ThomasParissi, VincentOrtego, JavierPark, EujinOrtner Hadziabdic, MajaPark, JunsooOscar D., SalomónPark, TaehwanO’Shea, JoyceParks, GriffithOsna, NataliaParolin, CristinaOsolodkin, DmitryParra, GabrielOSSIBOFF, Robert J.Parreira, RicardoOttis, EricaParrish II, Richard H.Ottmann, MichèleParrish, ColinOtulak-Kozieł, KatarzynaParrish, RichardOuwendijk, Werner J. D.Parsons, MatthewØynebråten, IngerPascal, Steven M.Ozawa, MakotoPascalis, HervéOzbun, MichellePasdeloup, DavidPacenti, MoniaPaskaleva, ElenaPacheco, BeatrizPassaes, Caroline Pereira BittencourtPacioni, CarloPassler, ThomasPaczesny, JanPastorek, JaromirPadhi, Bhaja KrushnaPatel, NibeditaPaes, Marciano VianaPatel, TejalPaeshuyse, JanPatel, Trushar R.Pagán, IsraelPathak, Vinay K.Page-Karjian, AnniePatil, BasavaprabhuPaillart, Jean ChristophePatman, ShanePaillot, RomainPatterson, StevenPaixão, Enny S.Patterson-West, JenniferPajkrt, DasjaPaulauskas, AlgimantasPalamara, Anna TeresaPaupy, ChristophePalinski, RachelPavelko, Kevin D.Palmenberg, Ann C.Pavesi, AngeloPalukaitis, PeterPavlov, KonstantinPammett, Robert T.Pawlak, AleksandraPandey, Krishan K.Paxton, RobertPayne, SusanPfeffer, MartinPazgier, MarzenaPhalen, DavidPearson, AngelaPhan, TungPECHEUR, EvePiancatelli, DanielaPeci, AdrianaPiantadosi, AnnePedersen, Finn SkouPiazza, RoccoPedrera, MiriamPichat, PierrePeeters, Ben P. H.Pierangeli, AlessandraPegan, Scott D.Pietersen, GerhardPei, JingjingPietrancosta, NicolasPei, YonggangPijlman, GorbenPeitsch, Manuel ClaudePikula, AnnaPelchat, MartinPilalas, DimitriosPelka, PeterPilotto, MarianaPena, Lindomar JoséPincus, SethPenades, JosePingarilho, MartaPeng, ChenPintel, DavidPenna, ClaudiaPinto, Amelia K.Pénzes, JuditPinto, Rosa MPerdoni, FedericaPipas, James M.Pereira, CarlaPires, CarlaPereira, Olívia R.Pires, DianaPereira, RobertoPischke, SvenPerelson, AlanPistello, MauroPérez Artés, EncarnaciónPitt, Michael BPerez Sepulveda, BlancaPittau, MarcoPerez, DanielPitter, Janos G.Pérez, Lester J.Pizzorno, MariePerez-Sautu, UnaiPizzuto, Maresca AttardPergadia, MichelePlattet, PhilippePerpelkin, JasonPleckaityte, MildaPersson, IngmarPlemper, RichardPeterlin, BorisPleshakova, Tatyana O.Peterson, GregoryPöhlmann, StefanPetraityte-Burneikiene, RasaPokorski, MieczyslawPetráš, MarekPolak, Mirosław PawełPetrides, K VPolonis, VictoriaPetrillo, MarcoPolyak, StephenPetrov, AntonPolz-Dacewicz, MałgorzataPetruzziello, ArnolfoPomar, LeóPetry, KlausPompon, JulienPetrzik, KarelPoncet, DidierPettersson, JohnPonraj, PrabakaranPettersson, UlfPonsero, Alise J.Peyambari, MahtabPonz, FernandoPfaender, StephaniePop, Tudor LucianPfaller, ChristianPopham, Holly J.Possee, RobertRagan, IzabelaPotapov, Sergey A.Raghavan, SudhirPoteat, ToniaRagonnert-Cronin, ManonPoterszman, ArnaudRagonnet-Cronin, ManonPotokar, MajaRagupathy, ViswanathPoulet, HervéRahman, MotiurPoulicard, NilsRai, Devendra KumarPouliopoulos, AntonisRaidoo, ShandhiniPoulson, RebeccaRainey, StephaniePowers, AnnRajão, Daniela S.Powers, James S.Rajarapu, Swapna PriyaPowis, Simon JohnRajarathnam, KrishnaPrabakaran, MookkanRajko-Nenow, PaulinaPradines, BrunoRakonjac, JasnaPrakasarao Kovvasu, SuryaRakotondrafara, AureliePrevelige Jr., Peter E.Ramachandran, RoshiniPreziuso, SilviaRamachandran, SujithPrice, PatriciaRamanathan, PalaniappanPrischi, FilippoRamette, AlbanPriyamvada, LalitaRamirez, HugoProdělalová, JanaRanadheera, SenakaProkhorov, NikolaiRanawade, AyushProsperi, AliceRandazzo, WalterProverbio, DanielaRappocciolo, GiovannaPuchol, Sandra MartínezRascher, WolfgangPuertas, Maria CarmenRashed, ArashPuff, ChristinaRashid, HarunorPuglisi, Melany P.Rasiah, RohanPuig-Barberà, JuanRastelli, EugenioPullan, Steven T.Rathinam, Navanietha KrishnarajPunga, TanelRathore, Abhay PSPuray-Chavez, Maritza N.Ratmann, OliverQin, ZhiqiangRausch, Jason W.Qiu, Hua-JiRauschnabel, Philipp A.Qu, BingqianRavelonandro, MichelQu, FengRavula, AbhigyanQuackenbush, SandraRay, RatnaQuadeer, Ahmed A.Raymond, AndreaQuéré, PascaleRayner, JonathanQuesada, ErnestoRazvan-Cosmin, PetcaQuigley, Bonnie L.Razzuoli, ElisabettaQuiñones-Mateu, Miguel E.Read, AndrewRabalski, LukaszRebollo, RitaRacine, TrinaRedd, AndrewRadke, Jay RReddy Bolla, JaniRadlinska, MonikaReddy, SanjayRadoshitzky, Sheli R.Reddy, VijayReddy, Vishwanatha R.A.P.Rivero-Juárez, AntonioRedwood, AlecRivkin, AnastasiaRegla-Nava, Jose A.Rizvanov, AlbertRegoes, RolandRobert, PhilippeReguera, JuanRoberts, JohnRegunath, HariharanRobinson, ChrisReichel, Michael P.Robinson, KarlReid, ScottRobinson, Sally R.Reid, St PatrickRoby, Justin A.Reid, StevenRocca, ElenaReid, WilliamRockett, Rebecca JReina, RamsésRockman, StevenReineke, LucasRodo, CarlotaReis, AnaRodriguez, FernandoRekkas, DimitriosRodríguez, FernandoReligioni, UrszulaRodriguez-Andres, JulioRen, PingRodríguez-Calleja, Jose MaríaResa-Infante, PatriciaRodríguez-Díaz, JesúsRety, StephaneRodriguez-Valera, FranciscoReuscher, CarinaRogier, BodewesRevill, PeterRohan, JenniferRevolinski, SaraRohrmann, George FReyes-Sandoval, ArturoRomalde, Jesús L.Reynolds, MaryRomano, Patricia SilviaRiccò, MatteoRomerio, FabioRichardson, Carolyn M.Ronca, ShannonRichardson, JuliaRong, Chan KuanRichman, Douglas D.Rong, LijunRichner, JustinRoot, Michael J.Richner, Justin M.Roque, FátimaRicht, JuergenRoques, PierreRied, L DouglasRosa, Bruce A.Riedel, ChristianeRosa, CristinaRieder, ElizabethRose, KarrieRieder, MichaelRose, OlafRiera, MartaRosenthal, MariaRigby, DebbieRosenthal, MeagenRigling, DanielRoss, PerranRiley, Kasandra J.Rossetto, AlyssiaRim, John HoonRossetto, Cyprian C.Rimstad, EspenRossi, GianpaoloRinder, MonikaRossier, OmbelineRisch, MartinRossor, ThomasRissanen, IlonaRost, GergelyRittschof, ClareRostal, MelindaRivas, CarmenRothenberger, SylviaRiveiro-Barciela, MarRothenburg, StefanRotondo, John CahrlesSaitou, NaruyaRoubalova, KaterinaSakai, HiroyasuRouillé, YvesSakamaki, IppeiRouse, MichaelSakellariou, DikaiosRousso, ItaySaksela, KalleRouth, AndrewSaláková, MartinaRouthu, Nanda KishoreSalama, Sofie R.Roux, SimonSalánki, KatalinRowland-Jones, SarahSalata, CristianoRowley, Paul AndrewSaleh, Fatima A.Royo, Luis J.Salman, M. D.Rozek, WojciechSalminen, OutiRuaro, BarbaraSalpini, RominaRubben, AlbertSalter, Jason D.Rückert, ClaudiaSalvato, MariaRudd, PennySalvetti, AnnaRudra, ArnabSamarasinghe, AmaliRueppell, OlavSamiotaki, MartinaRuggiero, GiuseppinaSamy, AhmedRuggli, NicolasSamy, RaviRuigrok, Rob W.H.Sánchez Pozo, AntonioRuiz-Garcia, Ana BelenSanchez, David JesseRuiz-Garcia, LeticiaSanchez, GloriaRumbou, ArtemisSanchez-Lockhart, MarianoRuml, TomasSánchez-Madrid, FranciscoRumlová, MichaelaSandro, RolesuRusanen, JuusoSang, YongmingRusconi, StefanoSangster, Mark Y.Russell, RodSankale, Jean-LouisRusu, AuraSanna, GiuseppinaRut, WiolettaSano, TeruoRutter, PaulSansone, PasqualeRyabov, EugeneSantiago-Rodriguez, Tasha MarieRybka, Jakub DaliborSantoro, Maria MercedesRynda-Apple, AgnieszkaSantos, RicardoRyndock, EricSantoso, NettyRyu, SangryeolSanz Muñoz, IvanSaad, JamilSapp, MartinSacco, Randy E.Sarathy, Vanessa V.Sadaoka, TomohikoSargueil, BrunoSaelens, XavierSaribaş, Abdullah SamiSafak, MahmutSarid, RonitSaha, IpsitaSariol, CarlosSahani, RajkumarSarker, SubirSahm, Laura JaneSarr, MoussaSahoo, Malaya KumarSarute, NicolasSaitoh, AkihikoSatheshkumar, Panayampalli SubbianSato, HironoriSchumpp, OlivierSattentau, QuentinSchvoerer, E.Sauder, ChristianSchwartz, David A.Saulle, IrmaSchwarz, AnkeSaura Lacárcel, José RamónSchweizer, MatthiasSauter, DanielSealy, JoshuaSautto, Giuseppe A.Sediva, AnnaSavenkov, EugeneSeed, SheilaSavic, VladimirSefat, FarshidSaxena, PoojaSeferovic, Maxim D.Scallan, MartinaSegal, EveScarborough, Robert J.Segalés, JoaquimScaturro, PietroSekiguchi, SatoshiSchachner, AdenaSekijima, MasakazuSchairer, CynthiaSeley-Radtke, KatherineScharf, BirgitSemino, CarlosSchemmerer, MathiasSemler, Bert L.Scheuermann, Richard H.Sencanski, MilanSchiattarella, AntonioSeo, Sang HeuiSchildgen, OliverSerra-Moreno, RuthSchildgen, OlivierServin, Argentina ESchindel, TerriSerwer, PhilipSchmidt, Aaron G.Setny, PiotrSchmidt, Peter ThelinSewald, KatherinaSchmidt, PhilipSewald, XaverSchmidtke, MichaelaSgouralis, IoannisSchneider, JenniferShackelford, JuliaSchneider-Schaulies, JuergenShaeer, Kristy M.Schneidman-Duhovny, DinaShah, PriyaSchnierle, Barbara S.Shahi, Shailesh K.Schoen, Robert T.Shakya, AkhileshSchols, DominiqueShamay, MeirSchommer, JonSharma, LokeshSchommers, PhilippSharma, ManviSchönrich, GüntherSharma, Narinder KumarSchotta, GunnarSharp, ClaireSchreiber, MichaelSharp, Natasha J.Schrier, RachelSharshov, KirillSchroeder, DeclanShaver, Amy L.Schrum, AdamShaw, AndrewSchubert, GritShaw, Margie HodgesSchubert, UlrichSheehy, NoreenSchuetz, AlexandraShepherd, AndrewSchuler Faccini, LaviniaSherer, NathanSchultz, DavidShestopalov, Alexander MSchultz, ThomasShi, MangSchulz, ClaudiaShiba, KiyotakaShih, ChiahoSlavcev, RoderickShim, HyunboSlimano, FlorianShimoda, MichikoŚliwińska-Wilczewska, SylwiaShimojima, MasayukiSloan, ChantelShimura, HanakoSluis-Cremer, NicolasShin, Hyun-JinSmartt, Chelsea T.Shin, JaekyuSmerdou, CristianShin, Ok SarahSmith, BruceShinkai, YoichiSmith, DonaldShioda, TatsuoSmith, DougShiokawa, MaiSmith, GregoryShirai, JyunsukeSmith, JessicaShirato, HarukoSmith, Lauren M.Shisler, JoannaSmith, Marie A.Shivanna, VinaySmith, MeganShpacovitch, VictoriaSmith, ThomasShriner, SusanSmith, Todd G.Shukla, DeepakSmithgall, Thomas E.Siamidi, AngelikiSmodiš Škerl, MajaSicheritz-Pontén, ThomasSmura, TeemuSiddappa, ByrareddySnoeck, Chantal J.Siddharthan, VenkatramanSnow, Jonathan W.Sieben, ChristianSöderberg-Naucler, CeciliaSignorini, LuciaSöderlund-Venermo, MariaSilva, RicardoSohn, SeonghyangSilva, WalterSokoloski, KevinSilveira, CynthiaSokolova, Olga S.Simeon, VittorioSolana, RafaelSimon, FrançoisSolis, MorganeSimpson, David J.Sollai, GiorgiaSimpson, MareeSolmaz, SozanneSin, JonSolovyev, AndreySinderewicz, EmiliaSompuram, SeshiSinganalur, Nagendrakumar BalasubramanianSon, KeunBaDaSingh, GurdeepSong, JianxunSingh, ReshmiSong, Min-SukSinigaglia, AlessandroSong, Moon JungSinkins, StevenSooryanarain, HariniSioofy-Khojine, AmirbabakSoriano, VicenteSirois, CarolineSoto, FernandoSironen, TarjaSoudeyns, HugoSistla, SrinivasSousa, AngelaSkelly, DeanneSouza, MeniraSkowron, AgnieszkaSozzi, EnricaSkurnik, MikaelSpada, EvaSlack, MarionSparger, ElizabethSlattum, PatriciaSpathis, Aris T.Spatz, StephenSturgill, MarcSpeck, Peter G.Su, WeihengSpengler, Jessica R.Suárez, Nicolás M.Spiegel, MartinSubbiah, JeevaSpiro, David J.Subr, ZdenoSpooner, S. AndrewŠubr, ZdenoSproule, BethSucena, ÉlioSridhar, SiddharthSuenaga, HikaruSt Hill, CatherineSuffredini, ElisabettaStadejek, TomaszSui, YongjunStafford, Graham P.Sukumaran, SunithaStainton, DaisySullivan, Brian M.Stamminger, ThomasSullivan, ChrisStämpfli, DominikSulzenbacher, GerlindStanifer, Megan L.Summers, MichaelStanton, James B.Sun, CaijunStapleford, KennethSun, HaiyanStapleford, Kenneth A.Sun, XiangjieStapleton, JackSun, XiaomingStavolone, LiviaSunderland, BruceStavrou, SpyridonSundram, SureshSteel, Laura F.Suomi, ReimaSteenhof, NaomiSurapathrudu, KanakalaSteger, GerhardSusi, PetriSteger, GertrudSutton, RichardSteinbach, FalkoSutummaporn, KripitchSteinke, DouglasSuzuki, TohruSteinmann, EikeSvircev, AntonetStepanova, EkaterinaSweeney, TrevorStephens, EdwardSwetnam, Daniele M.Sternberg-Lewerin, SusannaŚwiętoń, EdytaSTEVANOVIC, JevrosimaSwitzer, WilliamSteyer, Dr. AndrejSzacon, ElzbietaStobart, ChristopherSzakal, EvelinStone, DanielSzczepankiewicz, BarbaraStover, Kayla R.Szczepankowska, Agnieszka K.Stoye, Jonathan PaulSztanke, KrzysztofStrand, Micheline K.Szymczak, AleksandraStranges, Paul M.Tada, RuiStrawbridge, Judith D.Tagad, Harichandra D.Strecker, ThomasTai, WanboStrong, JimTai, William Chi-ShingStrutt, Tara M.Tajima, MotoshiStuart, PatrickTak, CaseyStubenrauch, FrankTakada, AyatoStukelj, MarinaTakahashi, TomokoStupans, IevaTakano, TomomiTakeda, ShigekiTessarz, PeterTakehira, RiekoTestoni, BarbaraTakemura, MasaharuThali, MarkusTakeuchi, RikiyaThanki, Anisha MahendraTakeuchi, YasuhiroTheuns, SebastiaanTakimoto, ToruThiebes, Anja LenaTakizawa, NaokiThiry, EtienneTalker, StephanieThomas, John C.Tamba, MarcoThomas, JulieTamori, AkihiroThompson, Amy N.Tan, WenbinThompson, Andrew J.Tanaka, YoshikazuThompson, JeremyTandon, RiteshThompson, JessicaTang, QiyiThursky, KarinTaniguchi, KokiThwaites, Ryan S.Taniguchi, SatoshiTian, YanpingTanniru, MohanTietjen, IanTao, PanTikoo, Suresh K.Tarasova, Ol’Ga A.Timofeev, Vladimir I.Tarlinton, RachaelTischer, KarstenTaskinen, SaraToborek, MichalTate, MichelleTodt, DanielTate, Shin-IchiToffan, AnnaTatsuya, KatoTognon, MauroTattersall, PeterTohma, KentaroTaube, StefanTollenaere, CharlotteTauriainen, SiskoTolstrup, MartinTaus, NaomiTomaszewski, DanielTavanti, FrancescoTompkins, ChristopherTaylor, AdamTonelli, MicheleTaylor, David G.Tonetti, Michela G.Taylor, IanTong, ShupingTaylor, JeffToniolo, Antonio Q.Te Velthuis, AartjanTonni, GabrieleTedbury, PhilipTonolo, GiancarloTelenitsky, AliceToplak, IvanTempera, ItaloTorcia, Maria GabriellaTempesta, MariaTorre, MarialuisaTemple-Smith, MeredithTorres, AliceTenenbaum, TobiasTortorella, DomenicoTeng, Lee LeeTorzewska, AgnieszkaTeramoto, TadahisaToth, KarolyTeras, RihoToubanaki, DimitraTerio, ValentinaToussaint, ArianeTerranova, CorradoTouzé, AntoineTerryn, SanneTrabaud, Mary-AnneTeschke, CarolynTrapp, SaschaTrapp-Fragnet, LaëtitiaUgaki, MasashiTravis, JohnUlaeto, DavidTraylor-Knowles, NikkiUlbert, SebastianTreder, KrzysztofUllah, Md AshikTremblay, NicolasUlrich, RainerTrinh, TrangUlrich, ReinerTripp, Ralph A.Umasuthan, NavaneethaiyerTrobaugh, DerekUnni, ElizabethTrotta, KatieUnni, Elizabeth J.Troyer, RyanUnzai, SatoruTrubl, GaryUpadhyay, ChitraTruncaitė, LidijaUpadhyay, KiranTruniger, VerónicaUpadhyay, SandeepTrus, IvanUpton, ChrisTsai, Chi-WeiUrayama, ShunichiTsai, Jih-JinUrbanelli, LorenaTsai, Ming-HanUrra, José MiguelTsetsarkin, Konstantin A.Usmani, ShariqTso, For YueUsui, TatsufumiTsourkas, PhilipposUversky, VladimirTsuge, HideakiV Ivanov, AlexanderTsukamoto, TetsuoVaidyanathan, RajivTsukiyama-Kohara, KyokoVaira, Anna MariaTsumoto, KantaVaismoradi, MojtabaTu, ThomasVales-Gomez, MarTucci, JosephValiakos, GeorgeTullman-Ercek, DanielleValkenburg, Sophie A.Tully, DamienValles, StevenTuma, RomanVan Baalen, MinusTumban, EbenezerVan Borm, StevenTun, Mya Myat NgweVan Bortel, WimTungadi, TrisnaVan De Klundert, MaartenTuomivirta, TeroVan De Vijver, David AMCTurchi, ChiaraVan Den Hoogen, BernadetteTurina, MassimoVan Den Hurk, AndrewTurnbull, Matthew W.Van Den Wollenberg, Diana J. M.Turner, DannVan Der Hoek, LiaTuru, GáborVan Der Kuyl, AntoinetteTwarock, ReidunVan Der Poel, WimTwigg, MichaelVan Der Sluis, Renée M.Tworowski, DmitryVan Der Velden, AlikeTyagi, MuditVan Dijk, LisetTyrrell, D LorneVan Doorslaer, KoenraadUcciferri, ClaudioVan Engelenburg, SchuylerUdoh, Arit EsioVan Etten, JamesUeda, KeijiVan Gils, Marit J.Uehara-Ichiki, TamakiVan Lint, CarineVan Marle, GuidoVolz, AsisaVan Matre, EdwardVon Dobschuetz, SophieVan Raaij, MarkVon Laer, DorotheeVan Rijn, PietVoroshilova, AnzhelikaVan Santen, VickyVosper, HelenVandeWoude, SueVranken, WimVaney, Marie-ChristineVu, LuanVanham, GuidoVujadinovic, MarijaVanlandingham, DanaVuorinen, TyttiVanpouille, ChristopheWadell, GöranVarallyay, EvaWagemans, JeroenVarjak, MargusWakimoto, HiroakiVarsani, ArvindWaldorf, Kristana AdamsVassaux, GeorgesWalker, Kathleen RVasse, MarcWalker, Peter J.Vassilaki, NikiWalkiewicz, KatarzynaVassilakos, NikonWalther, PaulVázquez-Domínguez, EvaristoWan, HongquanVecchio, EleonoraWan, WilliamVelasco, LeonardoWang, BoVelasco-Villa, AndrésWang, ChihyangVenuprasad, PoojaryWang, Ching-HoVerchot, JeanmarieWang, Chin-TienVernick, Kenneth D.Wang, DavidVérollet, ChristelWang, Fun-InVerrier, Eloi RWang, Hao-ChingViana, MafaldaWang, HURNG-YIVidana Mateo, BeatrizWang, JinViejo-Borbolla, AbelWang, JiongweiVijayan, MadhuvanthiWang, JuexinVilcek, StefanWang, JunVilibić-Čavlek, TatjanaWang, LeyiVilović, MarinoWang, LingVimalanathan, SelvaraniWang, NianshuangVinje, JanWang, RichardViranaicken, WildrissWang, Robert Y.-L.Visalli, Robert J.Wang, Shao-ChengVisseaux, BenoitWang, XiaohongVisser, Benjamin J.Wang, XiuqinVitour, DamienWang, YingVivet-Boudou, ValérieWang, ZhengqiangVlasova, Anastasia N.Wang, ZilongVogels, ChantalWang, ZiwenVogt, Peter K.Wang, ZongjieVolchkova, ValentinaWarner, NadiaVolle, RomainWarrell, MaryVolmer, DaisyWarrilow, DavidWasniewski, MarineWilson, Carolyn A.Watanabe, SatoruWilson, Van G.Watanabe, ShumpeiWilson, WilliamWatanabe, TokikoWilson, William C.Watkins, KimWilusz, JeffWebster, CraigWinkler, Michael E.Weger, StefanWinter, Deborah RachelleWegrzyn, AlicjaWira, CharlesWegrzyn, GrzegorzWise, Annabel G.Węgrzyn, GrzegorzWise, Emma L.Wei, DongqingWisniewski, Christopher S.Wei, HongpingWitkowski, WojciechWei, YonglongWitry, MatthewWeidner-Glunde, MagdalenaWold, William S.M.Weinbauer, MarkusWolf, StevenWeiser, RebeccaWölfel, RomanWeissenhorn, WinfriedWollebo, Hassen S.Weitzman, MatthewWong, GraceWelch, Stephen RWong, Richard W.Welsh, MichaelWong, Sek-ManWen, Chiu-MingWood, BrittaWenping, QiuWorkman, Aspen M.Wertheim, JoelWoźniakowski, GrzegorzWestman, MarkWright, DanielWettergreen, SaraWright, Daniel F. B.Weyenbergh, Johan VanWright, DavidWeynberg, KarenWu, AlanWhembolua, Guy-LucienWu, Fang-TzyWhitby, DeniseWu, GuanghuiWhite, JohnWu, I-ChinWhite, JudithWu, JianyongWhite, KirstenWu, Suh-ChinWhite, Peter A.Wu, WenchenWhite, SimonWu, WenxinWhitford, Paul CWu, XuelingWhitney, James B.Wu, YanlingWhitney, JohnWu, YuntaoWichgers Schreur, Paul J.Wylie, KristineWietholter, Jon P.Xavier, CatarinaWigdahl, BrianXia, NingshaoWilby, KyleXia, YuchenWilke, AndreXian, YuejiaoWilkes, Rebecca P.Xiang, XiaoqiangWilkinson, TraceyXiao, YongliWillett, BrianXie, DeyuWilliams, Charlene R.Xie, JiataoWilson, AngusXie, JiexiongXin, YinYoung, RylandXu, JianYount, Jacob S.Xu, PeiYousef, AhmedXu, WenxingYu, JingyouXue, GuangaiYu, Xiao-FangYamada, MitsuhikoYu, Xue-JieYamamoto, MasatoYuan, HaoYamamoto, TakehisaYuan, ShuofengYamamura, ShigeoYuan, XinxuYamanaka, AtsushiYuchun, DuYamaoka, SatokoYue, Xiao-GuangYamauchi, YoheiYuen, LillyYamayoshi, SeiyaYuh, Chiou-HwaYanase, TohruYuill, ThomasYang, Hung-ChihYura, YoshiakiYang, KuiZachou, KalliopiYang, SiyoungZago, MiriamYang, XiulingZaid, AliYao, YongxiuZakhartchouk, AlexanderYap, Kai ZhenZamperin, GianpieroYasuike, MotoshigeZanella, IsabellaYau, ShereeZanier, KatiaYe, XiaohuaZanusso, GianluigiYeager, MeredithZapata, JuanYeh, Hsin-HungZaslansky, RuthYehl, KevinZecchin, BiancaYellapu, Nanda KumarZeev-Ben-Mordehai, TzviyaYen, Pei-ShiZehender, GianguglielmoYeung, Eugene Y.H.Zeichner, Steven L.Yilmaz, Catalina Natalia CheaburuZell, RolandYin, JohnZeltins, AndrisYin, LiZeman, PetrYin, RenfuZeng, LanyingYin, ZhiminZgarrick, David P.Ylösmäki, ErkkoZhang, BoYolande, RichardZhang, DayiYoldi, Miguel Julián MartínezZhang, GongyiYoneyama, MitsutoshiZhang, Hong-YuYoo, Ji YoungZhang, HuanminYoo, So YoungZhang, QiongYoon, HachungZhang, RuiYoon, Sun-WooZhang, ShuoYoshida, EricZhang, WenliYoshimatsu, KumikoZhang, YinfengYost, Christian ConZhang, YongYouk, SungsuZhang, ZhenfengYoung, Howard A.Zhang, ZhidongZhang, ZhifangZhu, LinZhao, JianjunZhu, QiyunZhao, JunZhu, XueyongZhao, QinjianZidovec Lepej, SnjezanaZhao, Xue ZhiZienius, DainiusZheng, Du-PingZimmer, GertZheng, WenshuZimmerman, JeffreyZheng, XuexingZineldin, MosadZheng, ZhenhuaZmora, PawelZheng, Zhi-MingŻok, TomaszZheng, Zi-ZhengZoll, JanZhirnov, OlegZolnik, Christine P.Zhong, ZhaohuaZou, WeiZhou, AiminZumbrun, ElizabethZhou, DongmingŽunec, RenataZhou, Grace GZúñiga, ManuelZhou, Heshan SamZuñiga, SoniaZhou, PengZuo, YantaoZhou, ShuntaiZuriaga, ElenaZhu, JamesZüst, RolandZhu, JianZwick, Michael B.

